# Application of mRNA-Seq and Metagenomic Sequencing to Study *Salmonella pullorum* Infections in Chickens

**DOI:** 10.3390/ijms26041448

**Published:** 2025-02-09

**Authors:** Xiaohuan Chao, Zhexia Fan, Jiongwen Wu, Chutian Ye, Xiaomeng Wang, Ruina Li, Shuya Chen, Xiquan Zhang, Cheng Fang, Qingbin Luo

**Affiliations:** 1 State Key Laboratory of Livestock and Poultry Breeding, South China Agricultural University, Guangzhou 510642, Chinaxqzhang@scau.edu.cn (X.Z.); 2 College of Animal Science, South China Agricultural University, Guangzhou 510642, China

**Keywords:** *Salmonella pullorum*, chicken, DEGs, DEASs, microbial community

## Abstract

The disease caused by *Salmonella pullorum* has been demonstrated to exert a deleterious effect on the performance of poultry, giving rise to elevated mortality and considerable economic losses within the breeding industry. However, there is a paucity of research investigating the relationship between cecal gene expression and different isomer and *Salmonella pullorum* infection, and research on the relationship between intestinal microbiota and *Salmonella pullorum* infection is also limited. In this study, mRNA-Seq and metagenomic sequencing were performed on the cecal tissues and fresh feces of individuals who tested positive (*n* = 4) and negative (*n* = 4) for *Salmonella pullorum*, with the aim of exploring the chickens infected with *Salmonella pullorum* from two perspectives: the gene transcription level and the microbial level. The mRNA sequencing results revealed 1560 differentially expressed genes (DEGs), of which 380 genes were found to be up-regulated and 1180 genes were down-regulated. A number of genes were reported to be associated with immunity, including *AQP8*, *SLC26A3*, *CBS*, *IFI6*, *DDX60*, *IL8L1* and *IL8L2*. Furthermore, a total of 1047 differentially expressed alternative splicings (DEASs) were identified through alternative splicing analysis, including *CBS*, *SLC6A9*, *ILDR2*, *OCRL*, etc. The joint analysis of DEGs and DEASs revealed 70 genes that exhibited both differentially expressed alternative splicings and differential expression, including *CTNND1*, *TPM1*, *SPPL2A*, etc. The results of metagenomic sequencing demonstrated that the abundances of Bacteroides, Firmicutes, and Verrucobacteria underwent a significant alteration subsequent to the infection of *Salmonella pullorum*. In summary, the present study conducted a preliminary exploration of the genetic basis of chickens infected with *Salmonella pullorum*. *TPM1* and *SPPL2A* were found to be differentially expressed by mRNA-Seq, and differences in alternative splicing events. Furthermore, metagenomic sequencing revealed significant changes in the microbial communities of Bacteroidetes, Firmicutes, and Verrucobacteria during infection with *Salmonella pullorum*.

## 1. Introduction

The recessive white feather broiler demonstrates excellent production performance and hybridization advantages, which have played a significant role in the development of the high-quality chicken industry in China [1]. The serotypes of *Salmonella* enteritidis include *Salmonella typhi* and *Salmonella pullorum* [2]. While there are clear similarities between the two, the key difference is in the manner in which symptoms manifest and spread. Specifically, *Salmonella typhi* is primarily transmitted horizontally, whereas *Salmonella pullorum* is predominantly transmitted vertically [3,4]. *Salmonella pullorum* infection leads to a decrease in the laying rate and an increase in poultry mortality, resulting in significant economic losses for the breeding industry [5,6,7,8]. As demonstrated by Geng et al., the clinical signs exhibited by infected chicks within 21 days of age include white diarrhea, lethargy, dehydration, anorexia, and other symptoms, often accompanied by 100% mortality [9]. In adult chickens, the infection can result in a decline in egg production, hatching ability, and fertility. Furthermore, *Salmonella pullorum* has been observed to colonize the liver, spleen, and intestine for an extended duration, leading to adult chickens becoming recessive carriers [10,11].

It has been demonstrated that there are differences in the susceptibility and resistance of poultry to *Salmonella pullorum* in different genetic backgrounds and feeding environments [12]. Liu et al. demonstrated an association between polymorphisms in *MyD88* and susceptibility to *Salmonella pullorum* [13]. Furthermore, Setta et al. demonstrated that *Salmonella* infection with HD11 resulted in increased levels of *iNOS*, the pro-inflammatory cytokine *IL-6*, and the chemokines *CXCLi1* and *CXCLi2* [14]. Alternative splicing (AS) is a process that involves the production of different mRNA splicing isomers through various splicing methods, with the purpose of regulating gene expression [15,16]. Research has demonstrated that variable splicing plays a significant role in the development of colitis. Eden et al. have demonstrated that gene expression and splicing events are altered in long-term ulcerative colitis [17]. Sun et al. demonstrated that *SLC7A6-RI* isomeric intron knockdown promotes colon cancer cell proliferation [18].

*Salmonella pullorum* is a bacterium that typically infects poultry via the fecal–oral route. Following infection, the bacterium colonizes the digestive tract of the host, primarily the ileum and cecum, and causes systemic infection. The ileum is widely regarded as the primary site of immune activation, but *Salmonella enterica* serotype enteritidis has been found to colonize the cecum for a more protracted duration [19]. The cecum has been shown to be a more conducive environment for long-term colonization than the ileum [20]. It has been demonstrated that intestinal microbiota have the capacity to maintain the normal function of intestinal villi, to regulate the immune response, and to protect the host from pathogens and symbiotic bacteria [21,22,23,24]. The gastrointestinal tract of poultry is a vital immune organ of the host, comprising numerous innate immune cells [25]. The impact of environment, feed, and gastrointestinal microbiota on chicken intestinal health is well documented [26]. Wang et al. demonstrated that the regulation of intestinal microbiota can enhance poultry growth performance, reduce intestinal inflammation, and restore intestinal mucosal damage [27]. Alterations or instability of the microbiome and changes in its biodiversity are characteristic of many gastrointestinal and metabolic diseases [28].

The multifactorial nature of Pullorum disease underscores the necessity for a comprehensive understanding of its regulatory mechanisms and therapeutic interventions. A systematic investigation into the alterations in intestinal gene expression, diverse splicing isomers, and microbial communities may hold significant promise in elucidating its pathophysiology and potential therapeutic targets. This study utilized mRNA-seq and metagenomic sequencing to identify numerous DEGs, DEASs, and microbial communities that exhibited significant alterations. These findings provide a theoretical foundation for further investigation into the genetic regulatory mechanisms and therapeutic interventions for Pullorum disease.

## 2. Results

### 2.1. Different Expression of Genes in Cecal Tissues of Positive Group (P) and Negative Group (N) of Salmonella pullorum Infection

**Figure 1:**
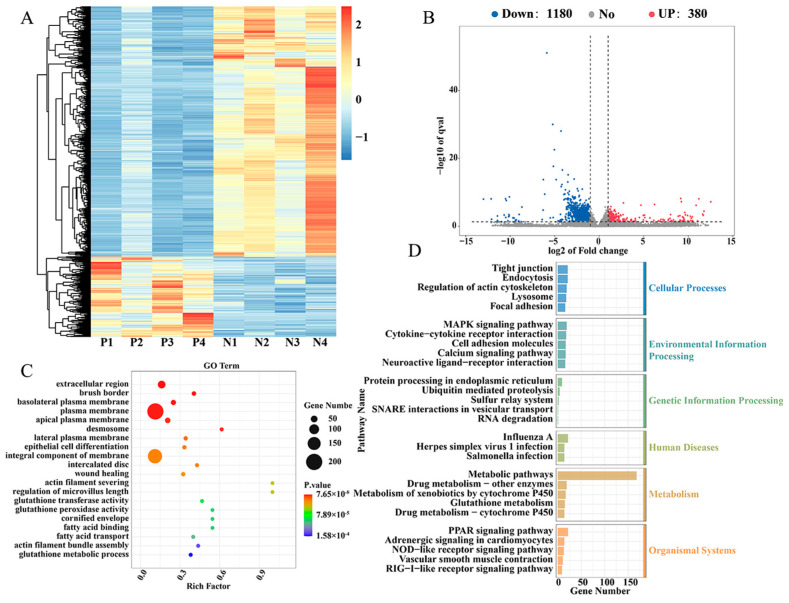
mRNA-seq analysis of cecal tissue in positive group (P) and negative group (N) of *Salmonella pullorum* infection. (**A**,**B**) Heat map and volcano plot for N vs. P DEGs. (**C**) GO function enrichment analysis for DEGs. X axis: gene number; Y axis: GO terms. (**D**) KEGG pathways classification analysis for DEGs. X axis: gene number; Y axis: pathway.

In order to investigate the genetic and molecular regulatory mechanisms potentially associated with *Salmonella pullorum* infection in recessive white feather broilers, a study was conducted in which the cecal tissues of the P with *Salmonella pullorum* infection (*n* = 4) and the N without *Salmonella pullorum* infection (*n* = 4) were subjected to transcriptome sequencing, and the results of the comparison between the sequencing data and the reference genomes are shown in Table S1. Utilizing the screening criteria of |log2FC| ≥ 1 and q < 0.05, 1560 DEGs were identified between the P and N groups, of which 380 genes were up-regulated and 1180 genes were down-regulated (Figure 1A,B). In order to explore the biological function of these DEGs, KEGG and GO enrichment pathway analysis was performed. GO enrichment analysis revealed that these DEGs were predominantly associated with protein binding, RNA transcription regulation, calcium ion binding, and gene expression regulation (Figure 1C). KEGG pathway enrichment analysis revealed that these DEGs were predominantly involved in metabolic signal transduction pathways, peroxisome proliferators-activated receptors (PPARs) signaling pathways, cell adhesion factors, and *Salmonella* infection (Figure 1D). These included *AQP8*, *SLC26A3*, *CBS*, *IFI6*, *DDX60*, *IL8L1*, *IL8L2*, etc. The top 20 differential genes are listed in Table 1.

### 2.2. Differentially Expressed Alternative Splicings of Cecal Tissue in Positive (P) and Negative (N) Groups of Salmonella pullorum Infection

**Figure 2:**
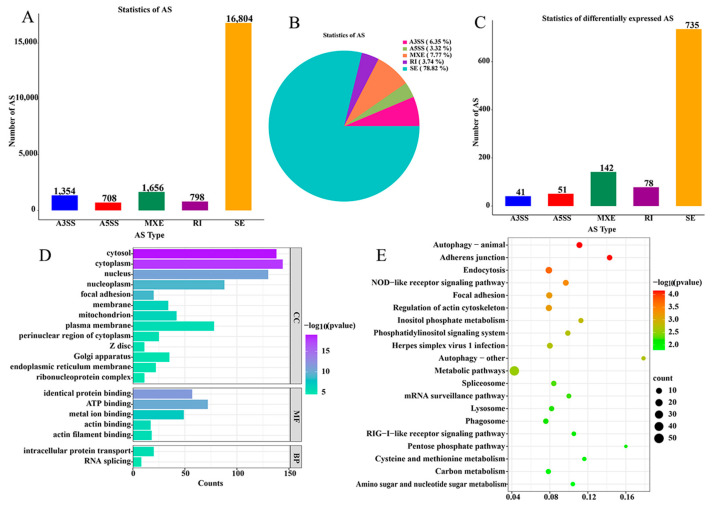
AS analysis of cecal tissue in the positive group (P) and the negative group (N) of *Salmonella pullorum* infection. (**A**) The following statistics were collated on the AS type, including A3SS, A5SS, MXE, RI, and SE. (**B**) The proportion of A3SS, A5SS, MXE, RI, and SE was counted. (**C**) DEASs in A3SS, A5SS, MXE, RI, and SE. (**D**) The GO function enrichment analysis for DEASs. (**E**) The KEGG pathways classification analysis for DEASs.

To investigate whether different transcripts are involved in the infection of *Salmonella pullorum*, we performed a variable splicing analysis of the mRNA data and found 21,320 ASs, of which 16,804 were SE, accounting for 78.82% of the total ASs (Figure 2A,B). All AS events were subjected to analysis using FDR < 0.05 and |IncLevel Difference| > 0.1 AS criteria for difference analysis. A total of 1047 ASs were found to be different, including 41 A3SS, 51 A5SS, 142 MXE, 78 RI, and 735 SE. (Figure 2C). The 1047 AS event was observed in 821 genes, which were subsequently analyzed using gene GO and KEGG. The results of GO and KEGG analyses showed that they were mainly enriched in cytoplasmic, cytosolic, and metabolic pathways (Figure 2D,E). Among them, genes related to immunity and inflammation are *CBS*, *SLC6A9*, *ILDR2*, *OCRL*, etc.

### 2.3. Combined DEGs and DEASs Analysis of Cecal Tissue from Positive (P) and Negative (N) Groups Infected with Salmonella pullorum

**Figure 3:**
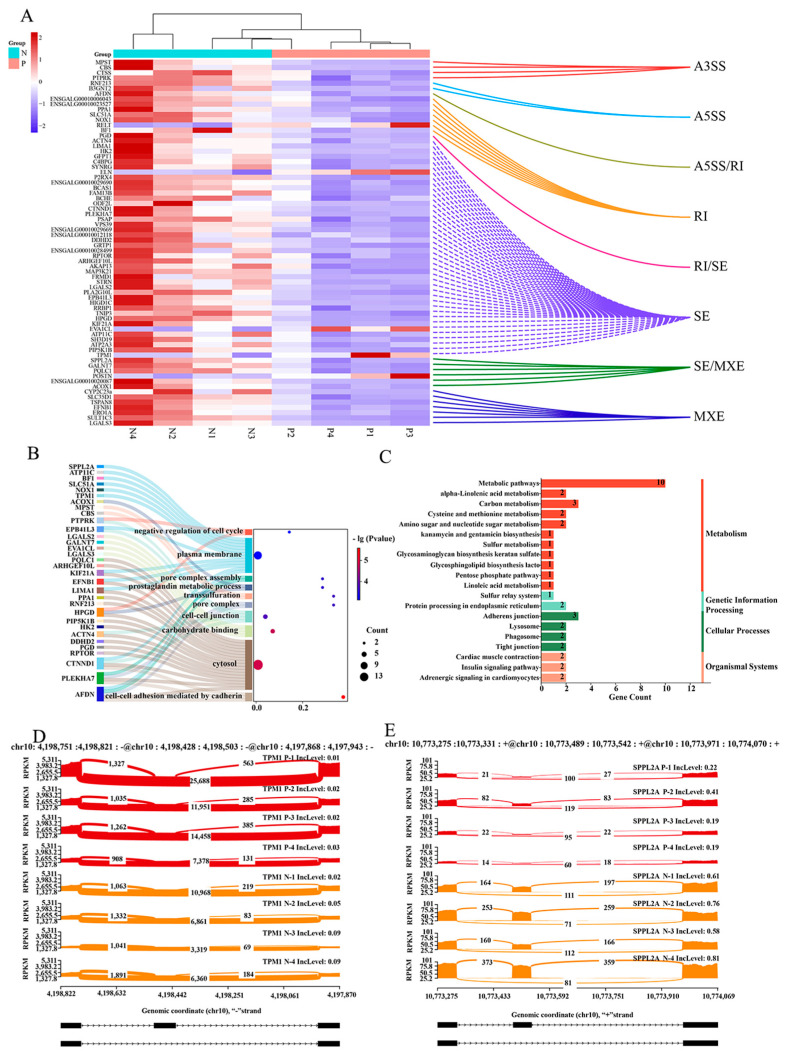
A combined analysis of DEGs and DEASs. (**A**) A heat map of differentially expressed and AS genes and their AS types. (**B**,**C**) The GO and KEGG pathways enrichment analyses for differentially expressed and AS genes. (**D**,**E**) The visual presentation is intended to demonstrate the phenomenon of differential AS on *TPM1* and *SPPL2A*.

In order to further understand the changes of DEGs and DEASs in P and N groups after *Salmonella* infection, a joint analysis of DEGs and DEASs was performed. The analysis yielded results that indicated that 80 alternative splicing (AS) events were identified in 70 genes, and these ASs and gene expressions were simultaneously different. (Figure 3A). Subsequent GO and KEGG analyses of these 70 genes revealed that they were predominantly enriched in pathways related to the plasma membrane, cytoplasm, metabolism, and other processes (Figure 3B,C). These included *CTNND1*, *TPM1*, *SPPL2A*, etc. In this way, we visualized AS events with a high abundance of *TPM1* and *SPPL2A* (Figure 3D,E).

### 2.4. The Metagenomic Sequencing of Cecal Tissue Was Conducted in the Positive (P) and Negative (N) Groups Infected with Salmonella pullorum

**Figure 4:**
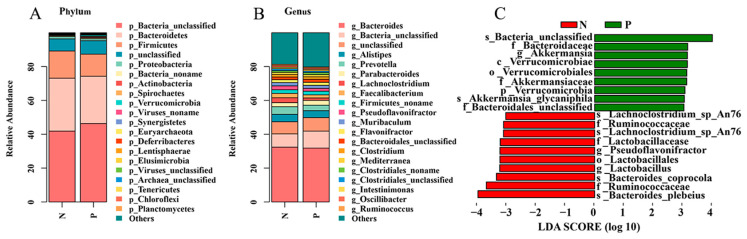
Microbiological analysis of *Salmonella pullorum* infection in the P group and the N group. (**A**) The top 20 species in terms of phylum-level abundance. (**B**) The top 20 species in terms of genus-level abundance. (**C**) LEfSe analyzed biomarkers that were significantly different in the *Salmonella pullorum* infection N and P groups.

In order to further explore the changes in the intestinal microbiota in infected chickens after *Salmonella pullorum* infection, the metagenomic sequencing of fresh feces was performed on individuals in the P (*n* = 4) and N groups (*n* = 4). A total of 50.15 G of original data was obtained, and 40.36 G of effective data was obtained after pretreatment (Table 2). A composition analysis of the metagenomic data found that, at the gate level, the relative abundance of Actinobacteria (0.47% vs. 0.36%), Spirochaetes (0.45% vs. 0.30%), Synergistetes (0.27% vs. 0.23%), Euryarchaeota (0.26% vs. 0.14%), and Lentisphaerae (0.12% vs. 0.02%) was higher in the P group. In contrast, the N group exhibited a higher relative abundance of Bacteroidetes (31.27% vs. 27.92%), Firmicutes (16.05% vs. 13.03%), Proteobacteria (1.34% vs. 1.14%), and Deferribacteres (0.11% vs. 0.04%) (Figure 4A). At the genus level, g_Bacteroides and g_Akkermansia were more abundant in the P group. Conversely, the abundance of g_lactobacillus and g_pseudoflavonifractor was higher in the N group (Figure 4B,C). In order to distinguish biomarkers with significant abundance differences between positive and negative groups, LEfSe analysis was performed for the two groups. The LEfSe analysis revealed that Verrucomicrobia, Bacteroidaceae, and Akkermansia were more abundant in the P group. Conversely, Lactobacillus and Pseudoflavonifractor were abundant in the N group (Figure 4C).

## 3. Discussion

The highly infectious nature of the disease caused by *Salmonella pullorum* has a significant impact on performance and survival rates in the chicken production industry [6,8,29]. The current approach to purifying pullorum disease from the actual production involves the elimination of infected individuals. In this study, we collected cecal tissues from positive individuals (*n* = 4) and negative individuals (*n* = 4) to explore candidate genes and pathways associated with white dysentery infection in chickens at the transcriptome level. The KEGG enrichment analysis of DEGs revealed that these DEGs were predominantly enriched in metabolic signal transduction pathways, PPAR signaling pathways, cell adhesion factors, and *Salmonella* infection. Among them were *AQP8*, *SLC26A3*, *CBS*, *IFI6*, *DDX60*, *IL8L1*, *IL8L2,* and other candidate genes. The *SLC26A3* protein plays an important role in maintaining the intestinal mucosal barrier, controlling intestinal inflammation, maintaining a stable intestinal pH value, and regulating intestinal flora [30,31,32]. *SLC26A3* expression is decreased in inflammatory bowel disease. Kini et al. demonstrated that in *SLC26A3* knockout mice, the cecum, colon, and mucosal adhesion flora were abnormal, with a loss of diversity, increased short-chain fatty acid production, and increased levels of pathogenic bacteria [33]. This result is consistent with the present study, in which *SLC26A3* expression was reduced after infection with *Salmonella pullorum* infection, and beneficial bacteria were significantly reduced in the *Salmonella pullorum* infection group. *IL8*, a chemokine, has been demonstrated to play a pivotal role in the processes of inflammation and immune response within poultry species [34,35]. This assertion is further substantiated by the findings of Vu et al., which revealed that the expression level of *IL8* in H5N1-resistant chickens exceeded that observed in H5N1-sensitive chickens [36]. Studies have demonstrated the pivotal role of cytokines as signaling molecules in the host’s defense against enteric pathogens, such as *Salmonella*. Following infection with *Salmonella pullorum*, there is an increase in *IL8* expression within the host [37]. Furthermore, Swaggerty et al. demonstrated increased mRNA expression levels of proinflammatory cytokines *IL6*, *IL8*, and *IL18* in individuals infected with *Salmonella pullorum*, despite the presence of only limited inflammatory infiltration [10,38]. This finding is consistent with the results of our study, which demonstrated a reduction in *IL8* mRNA levels in chickens infected with *Salmonella pullorum*.

The application of alternative splicing analysis of mRNA data yielded the identification of 1047 distinct AS events in the cecum tissue of the P group and the N group. Furthermore, through the joint analysis with DEGs, it was found that 70 genes were not only differentially expressed but were also differentially spliced. These included *CTNND1*, *TPM1*, and *SPPL2A*, amongst others. Research has demonstrated that *TPM1* generates multiple isomers through variable splicing and executes distinct functions under varying physiological and pathological conditions [39,40,41]. Gardina et al. demonstrated that there is a discrepancy between the AS of *TPM1* colon cancer tumor tissue and normal colon tissue and that the isomer produced by the variable splicing is more highly expressed in colon cancer tumor tissue [42]. In this study, *TPM1* IncLevel was found to be diminished in the P group, suggesting that exon jumps and the production of isomers are increased. This finding is in accordance with the results reported by Gardina et al.

Metagenomic sequencing analysis revealed significant changes in microflora abundance. Research has demonstrated that *Salmonella* has the capacity to destroy the dominant beneficial bacteria in the gut and modify the colonization of the host intestinal flora. This, in turn, results in the ability to resist the host’s normal intestinal flora and immune response, thereby creating a disadvantage for the normal intestinal flora [43,44,45]. Huang et al. demonstrated that *Salmonella* infection significantly reduced the relative abundance of Lachnoclostridium and Blautia, while phage *CKT1* treatment significantly increased the proliferation of beneficial microflora in intestinal Firmicutes [46]. These included Lachnoclostridium, Ruminococcus, Lactobacillus, and pseudoflavonyifrtor. This finding is further corroborated by the study conducted by Ding et al., which demonstrated that the abundance of lactobacillus in the N group of *Salmonella pullorum* infected is higher than that in the P group [47]. This finding is corroborated by the present study, which demonstrates that the abundance of Firmicutes in the N group exceeds that of the P group, and that the abundance of Lactobacillus and Pseudoflavonifractor is higher in the N group.

## 4. Materials and Methods

### 4.1. Experimental Animal Management

In this study, subwing vein blood was collected from 100-day-old fourth-generation recessive white feather broilers. These chickens were bred by Guangzhou Jiangfeng Industrial Co., Ltd. (Guangzhou, Guangdong, China). A plate agglutination test was then performed to identify individuals infected with *Salmonella pullorum*. Subsequent to this, fresh feces were subjected to PCR, and the results of this analysis are presented in Figure S1. Individuals with plate agglutination results that were completely consistent with the PCR results were designated as positive or negative, and a total of eight individuals were euthanized. The cecum and its contents were then collected and placed in liquid ammonia, after which they were transported via dry ice to LC-Bio Technologies Co., Ltd. (Hangzhou, Zhejiang, China) for mRNA-seq and metagenomic sequencing, respectively. The animal experiment was approved by the Animal Health Professional Committee of South China Agricultural University (SCAU#2017015; 13 September 2017).

### 4.2. Serum Plate Agglutination Test

Subwing blood sampling was performed on 100-day-old recessive white feather broilers hens. Following the collection of blood, it was left to precipitate serum. Thereafter, serum and antigenic reagent (YDLH042, Shanghai, China) were added in a ratio of 1:1. The mixture was then shaken at room temperature for a period of 2–5 min. The presence of more than 50% agglutination was taken to indicate a positive result. In the absence of agglutination, a negative result was recorded. When the agglutination was observed to be between the two extremes, the result was deemed to be suspicious (Figure S2).

### 4.3. PCR Experiment

The extraction of deoxyribonucleic acid (DNA) was conducted utilizing a fecal DNA extraction kit (CW2091S, Shanghai, China). The subsequent PCR reaction was performed in strict accordance with the product instructions of the 2×Taq PCR MasterMix (B639295, Shanghai, China). The primer information is in Table S2.

### 4.4. mRNA-Seq of Cecal Tissues and Alternative Splicing Analysis

The total RNA was extracted from cecal tissues using TRIzol (Thermofisher,, CA, USA) according to the manufacturer’s instructions. The quantity and purity of the total RNA were then measured using a NanoDrop ND-1000 (NanoDrop, Wilmington, DE, USA) at Unikawa Bio, after which the RNA was analyzed using a Bioanalyzer 2100 (Agilent, CA, USA). The integrity of the RNA was detected, and mRNA sequencing was completed. Alternative splicing was detected using rMAST (rmats4.1.2), and the KOBAS software (http://bioinfo.org/kobas, accessed on 1 February 2025) was utilized to detect the enrichment of the Gene Ontology (GO) and Kyoto Encyclopedia of Genes and Genomes (KEGG) pathways in the differential genes [48]. This process was completed by LC-Bio Technologies Co., Ltd. (Hangzhou, Zhejiang, China).

### 4.5. DNA Extraction and Metagenomic Sequencing

The otal DNA was extracted from microbiome samples using the CTAB method. NovaSeq6000 was used for high-throughput sequencing, and the original sequencing data were assembled. MetaGeneMark (v3.26) was then used for CDS prediction, after which the predicted results were clustered. The reads of each sample were then compared to the CDS sequence library for TPM abundance calculation and species annotation information. Function annotation was achieved through comparison with various databases, including eggNOG, CAZy, CARD, PHI, MGEs, and VFDB. A statistical analysis of abundance and a comparative analysis of differences were performed at the species, function, and gene levels. This process was completed by LC-Bio Technologies Co., Ltd. (Hangzhou, Zhejiang, China).

## 5. Conclusions

In summary, in our study mRNA sequencing was utilized for the screening of candidate genes associated with *Salmonella pullorum* infection, including *AQP8*, *SLC26A3*, *CBS*, *IFI6*, *DDX60*, *IL8L1*, *IL8L2*, and others. Alternative splicing analysis identified the splicings associated with *Salmonella pullorum* infection, including *CBS*, *SLC6A9*, *ILDR2*, *OCRL*, *CTNND1*, *TPM1,* and *SPPL2A*. Metagenomic sequencing revealed that the relative abundance of p-verrucomicrobia increased and the relative abundance of p-Firmicutes decreased when chickens were infected *Salmonella pullorum*. The present study screened out candidate genes, AS isomers, and microbiota associated with *Salmonella pullorum* infection, thus providing a theoretical basis for the study of the regulatory mechanism and treatment of *Salmonella pullorum* infection. In subsequent studies, efforts should be made to restore the balance of beneficial microbes through targeted interventions, or by applying specific RNA editing techniques. This approach has the potential to offer a novel direction for the treatment and prevention of *Salmonella pullorum* infection.

## Figures and Tables

**Table 1:** The top 20 genes were differentially expressed in the negative group and positive group of *Salmonella pullorum* infection.

Gene Name	N Expression	P Expression	log2(FC) (N/P)	Up Down
*AQP8*	8631	146.75	−5.875936928	down
*ENSGALG00010017812*	10,723.5	298	−5.212645245	down
*RSAD2*	2291.75	118	−4.288236383	down
*ENSGALG00010001707*	45,105.25	1414.75	−5.028950395	down
*CBS*	94,320.75	2736.25	−5.147365626	down
*ENSGALG00010021763*	4732.75	269	−4.133430962	down
*ENSGALG00010005012*	2873.25	257.5	−3.485079358	down
*SCNN1A*	4646.25	674.25	−2.809438512	down
*DDX60*	5710	206	−4.830147732	down
*ENSGALG00010017719*	517	7.5	−6.278950066	down
*ANPEP*	20,453.25	1817.75	−3.531739751	down
*SLC26A3*	1882	104.75	−4.179327946	down
*IFI6*	18,342	986.5	−4.269812813	down
*EDN2*	532.75	98	−2.453229646	down
*COBL*	1703.5	463.5	−1.897709004	down
*SLC30A10*	1501.25	111.75	−3.790121129	down
*EPSTI1*	2068	406.25	−2.365929024	down
*SERPINB1*	3981.75	401.5	−3.35058651	down
*LAMC2*	1558.75	138	−3.512285305	down
*KRT40*	22,899.5	1603	−3.858303875	down

**Table 2:** The metagenomic sequencing data of *Salmonella pullorum* infection positive group and negative group.

Sample	Original Data (G)	Valid Data (G)	Valid Data Ratio (%)	Q20%	Q30%	GC%
P1	6.53	5.45	89.52	98.56	95.52	52.21
P2	6.35	5.32	90.04	98.50	95.43	51.18
P3	6.48	5.29	88.33	98.49	95.43	50.28
P4	6.34	5.16	88.02	98.51	95.49	52.07
N1	5.31	4.26	87.14	98.64	95.81	52.06
N2	6.21	4.72	83.50	98.59	95.70	51.47
N3	6.44	5.05	85.86	98.58	95.71	51.62
N4	6.49	5.11	85.72	98.69	95.88	51.20

## Data Availability

The data sets that have been either generated or analyzed during the course of this study are available for acquisition from the corresponding authors.

## Supplementary Materials

The following supporting information can be downloaded at: https://www.mdpi.com/article/10.3390/ijms26041448/s1.

## Abbreviations

The following abbreviations are used in this manuscript:

DEGs	Differentially expressed genes
DEASs	Differentially expressed alternative splicings
P group	positive group
N group	negative group
AS	Alternative splicing
