# Supplementary material for: Application of mRNA-Seq and Metagenomic Sequencing to Study Salmonella pullorum Infections in Chickens
Source: Int J Mol Sci. 2025 Feb 9;26(4):1448. doi: 10.3390/ijms26041448 (PMC11855712; doi:10.3390/ijms26041448)
Supplement: Supplementary file 1 [file ijms-26-01448-s001.zip › ijms-3454263-supplementary.pdf]

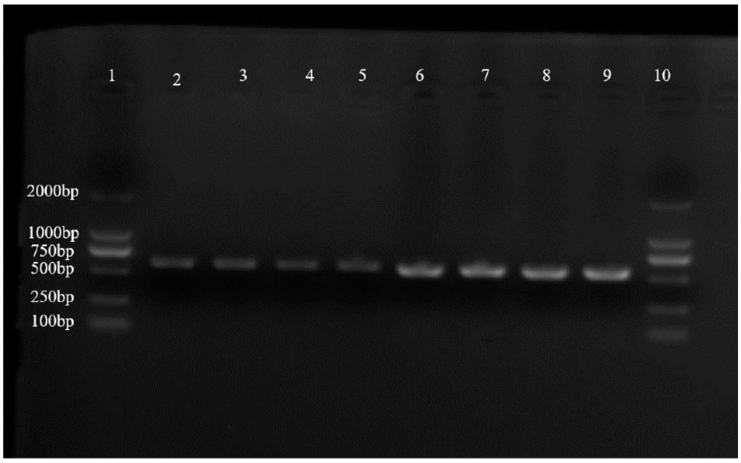

Figure S1. Individuals identified by PCR as positive: Lane 1 and 10 were Maeker lanes, while lanes 2, 3, 4 and 5 contained positive individual samples. Lanes 6, 7, 8 and 9 contained standard samples of Salmonella pullorum.

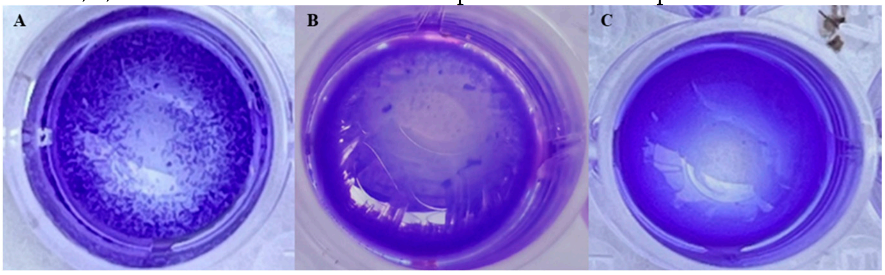

Figure S2. Salmonella pullorum infection positive individual and negative individual determination: (A) Judged as a positive individual. (B) Judged as a negative individual. (C) Judged as a suspicious individual.

Table S1. Statistical table of results of comparison between sequencing data and reference genome sequence.

Sample	Raw Data	Valid Data	Valid Data Q20 Rate	Valid Data Q30 Rate
P1	42879698	39261590	99.93	97.90
P2	39840940	36953124	99.94	97.83
P3	42655208	38411186	99.93	97.84
P4	44060278	37768118	99.94	98.01
N1	44499392	41449186	99.94	97.78
N2	40023508	37258792	99.93	97.78
N3	40482114	37754522	99.94	97.90
N4	39135674	36506124	99.94	97.89

Table S2. The primer pairs information of related gene.

Gene	Primer sequence(5′→3′)	Product size (bp)
SEEP400405-F	GAGAATCCGGGACGGATGAC	576
SEEP400405-R	CACTCGACAGGAACGCATTG	
